# Optimal Strategy on Radiation Estimation for Calculating Universal Thermal Climate Index in Tourism Cities of China

**DOI:** 10.3390/ijerph19138111

**Published:** 2022-07-01

**Authors:** Jiandong Liu, Guangsheng Zhou, Hans W. Linderholm, Yanling Song, De-Li Liu, Yanbo Shen, Yanxiang Liu, Jun Du

**Affiliations:** 1 State Key Laboratory of Severe Weather, Institute of Agro-Meteorology and Ecology, Chinese Academy of Meteorological Sciences, Beijing 100081, China; liujd2001@263.net (J.L.); songyl@cma.gov.cn (Y.S.); 2 Department of Earth Sciences, University of Gothenburg, 405 30 Gothenburg, Sweden; hans@gvc.gu.se; 3 Department of Geography, University of Cambridge, Cambridge CB2 3EN, UK; 4 NSW Department of Primary Industries, Wagga Wagga Agricultural Institute, PMB, Wagga Wagga, NSW 2650, Australia; de.li.liu@dpi.nsw.gov.au; 5 Climate Change Research Centre, University of New South Wales, Sydney, NSW 2052, Australia; 6 Public Meteorological Service Centre, China Meteorological Administration, Beijing 100081, China; shenyb@cma.gov.cn (Y.S.); liuyx@cma.gov.cn (Y.L.); 7 Tibet Institute of Plateau Atmospheric and Environmental Science Research, Tibet Autonomous Meteorological Administration, Lhasa 850001, China; dujun0891@163.com

**Keywords:** UTCI calculation, radiation estimation, thermal comfort, public health

## Abstract

The Universal Thermal Climate Index (UTCI) is believed to be a very powerful tool for providing information on human thermal perception in the domain of public health, but the solar radiation as an input variable is difficult to access. Thus, this study aimed to explore the optimal strategy on estimation of solar radiation to increase the accuracy in UTCI calculation, and to identify the spatial and temporal variation in UTCI over China. With daily meteorological data collected in 35 tourism cities in China from 1961 to 2020, two sunshine-based Angstrom and Ogelman models, and two temperature-based Bristow and Hargreaves models, together with neural network and support vector machine-learning methods, were tested against radiation measurements. The results indicated that temperature-based models performed the worst with the lowest *NSE* and highest *RMSE*. The machine-learning methods performed better in calibration, but the predictive ability decreased significantly in validation due to big data requirements. In contrast, the sunshine-based Angstrom model performed best with high *NSE* (Nash–Sutcliffe Efficiency) of 0.84 and low *RMSE* (Root Mean Square Error) of 35.4 J/m^2^ s in validation, which resulted in a small *RMSE* of about 1.2 °C in UTCI calculation. Thus, Angstrom model was selected as the optimal strategy on radiation estimation for UTCI calculation over China. The spatial distribution of UTCI showed that days under no thermal stress were high in tourism cities in central China within a range from 135 to 225 days, while the largest values occurred in Kunming and Lijiang in southwest China. In addition, days under no thermal stress during a year have decreased in most tourism cities of China, which could be attributed to the asymmetric changes in significant decrease in frost days and slightly increase in hot days. However, days under no thermal stress in summer time have indeed decreased, accompanying with increasing days under strong stress, especially in the developed regions such as Yangze River Delta and Zhujiang River Delta. Based on the study, we conclude that UTCI can successfully depict the overall spatial distribution and temporal change of the thermal environments in the tourism cities over China, and can be recommend as an efficient index in the operational services for assessing and predicting thermal perception for public health. However, extreme cold and heat stress in the tourism cities of China were not revealed by UTCI due to mismatch of the daily UTCI with category at hourly scale, which makes it an urgent task to redefine category at daily scale in the next research work.

## 1. Introduction

There is a close relationship between human thermal perception and the atmospheric environment [1], but humans do not have receptors to sense the air temperature directly [2]. Rather, what humans feel in the daily experience is actually the comprehensive summary of the thermal environmental conditions, such as skin temperature and their demand for heating or cooling [3], which are influenced directly or indirectly by the atmospheric elements such as solar radiation, air temperature, humidity, and wind speed etc. [3,4,5,6]. Thus, thermal comfort index, rather than simple air temperature, has drawn increased attention in the recent decades, in order to provide better services for assessing and predicting thermal perception in the domain of tourism, public health, and climate impact assessment [7,8,9,10].

Initially, many two-parameter indices were developed to represent the human thermal environment, including the effective temperature [11], the wind chill index [12], and the temperature–humidity index [13]. Though these empirical indices can be easily calculated with simple algorithms, they neglect significant variables and fluxes influencing thermal perception, which would inevitably lead to misrepresentation of the thermal environment [7]. In the recent decades, many heat budget models have been developed in the field of thermal biometeorology, including representatives such as the MEMI model [14], the Klima–Michchel Model [1], and the MENEX model [15]. All of these heat budget models can be used in the assessment of the thermal environment, but none is accepted as the fundamental standard due to persistent shortcomings caused by the relevant theory on heat exchange and thermos-physiology [7]. Finally, through the cooperation by scientists from many countries, the Universal Thermal Climate Index (UTCI) was established under the commission supposed by the International Society on Biometeorology [3,7,16].

Based on the achievements in many previous heat budget models, especially the Fiala model, UTCI has fully considered the comprehensive influence of the atmospheric environment on human perception [3,17,18]. Up to now, UTCI has been validated extensively with measured data from climate chamber or wind tunnel experiments, together with data collected from outdoor surveys [3,19]. It has been identified that UTCI is sensitive to small variations in the atmospheric environment [20], and believed to be suitable for assessing thermal environments under all climate conditions [7,21,22,23,24,25,26].

However, everything has two sides. While UTCI has great advantage over the simple empirical indices in describing thermal perception due to its full consideration of the atmospheric environment, this consideration inevitably has led to the negative influence on its application caused by its requirements of more input meteorological variables [3,4,16,27], among which solar radiation is certainly more difficult to access than the other meteorological items such as temperature and humidity etc. Under this condition, cloudiness was often used as proxy of solar radiation as the input variable [9,23,24,28], but the reliability of the UTCI results might be suspicious, as calculation by cloudiness might result in high discrepancy in radiation estimation [9].

In fact, solar radiation can be accurately estimated by many methods, including robust numerical models [29,30], remote sensing [31,32], and empirical models [33,34,35]. In recent decades, empirical models, including sunshine- and temperature-based models, have become popular in radiation estimation due to their simple operation and readily available variables [36,37]. The sunshine-based models are more preferable to temperature-based ones because of its high accuracy in radiation estimation [33,38], even in the extreme climate regions such as the Tibetan Plateau [39]. Recently, many machine-learning methods have also been used to estimate solar radiation, and have shown great promise with their high accuracy [40,41]. Based on the sensitivity analysis, Weihs et al. [4] argued that the uncertainty in the calculated UTCI might be less than ±2 °C; if radiation was reasonably estimated with synoptic observations, so we can envisage that accurate UTCI can be obtained based on optimal strategy on estimating solar radiation with empirical models or machine-learning methods. However, to our knowledge, no research has yet has been conducted to test this hypothesis.

China covers a huge area with complex topography and diverse climate [42], which highlights the importance of the assessment of its thermal environment for public services [9]. In recent years, some studies on UTCI have already been performed to provide information on the thermal environment over China [9,10,28,42,43]. However, like the research conducted in the other countries mentioned above [21,22,23,24,25], UTCI also cannot be easily calculated in China due to paucity of solar radiation observation, as there are only about four percent (100 out of 2500) weather stations routinely observe solar radiation over China, due to scarcity of radiation instruments and their high costs of maintenance [38,41]. Under this circumstance, UTCI in China was often calculated by some readily available items such as cloudiness [9,28,43], or estimated by simple spatial interpolation of solar radiation [44], or even analyzed by avoiding the input requirement of solar radiation with assumption that radiative temperature just equals air temperature [10].

In this study, the sunshine-based Angstrom and Ogelman models, and the temperature-based Bristow and Hargreaves models, together with neural network and support vector machine-learning methods, were used to estimate solar radiation for calculating UTCI in 35 tourism cities of China. The objectives of this study were: (1) to investigate the influence of different strategies of radiation estimation on the accuracy of UTCI calculation, based on which the optimal strategy could be identified; (2) to provide spatial distribution of UTCI in tourism cities over China; and (3) to reveal the temporal trend in thermal stress in these cities in the recent 60 years, which would be beneficial for the local governments to make relevant policies on assessing and predicting thermal perception for the public health. 

## 2. Materials and Methods

### 2.1. Database

**Figure 1:**
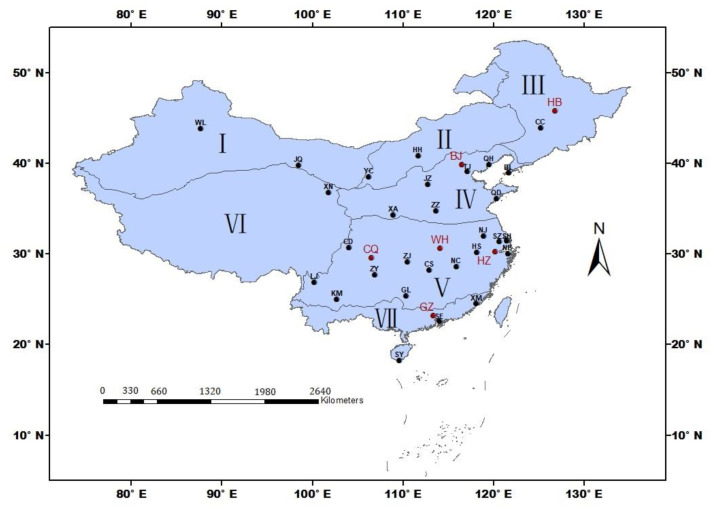
Distribution of the large tourist cities in this study. The cities with red color are used for exploring optimal methods on solar radiation estimation, and the cities with black color are the other tourism cities in this study. Roman numerals indicate the climate zones in China. I denotes the temperate and warm-temperate deserts of northwest China, II Inner Mongolia, III the temperate humid and sub-humid northeastern China, IV the temperate humid and sub-humid northern China, V the subtropical humid central and southern China, VI the Qinghai–Tibetan Plateau, and VII the tropical humid southern China. Whole names of the cities can be seen in Table 1.

Chinese main tourism destinations, including 35 cities distributed in different climate zones (Figure 1), were selected according to the classification based on comprehensive development indices of tourism industry, urbanization, and ecological environment [45]. Detailed information on these tourism cities can be seen in Table 1.

For this research, access to the fundamental database of NMIC (National Meteorological Information Center) was given by CMA (China Meteorological Administration). The daily meteorological data, including sunshine hours, mean temperature, maximum temperature, minimum temperature, vapor pressure, relative humidity, wind speed, precipitation, and cloudiness, were collected from 1961 to 2020 for all of these locations. There were very few missing values in the dataset. When a missing value was identified, it was substituted by the average of the values observed on preceding and following days [10]. Solar radiation is not a routinely observed meteorological item in most weather stations in China, and the daily solar radiation data were only available in six stations, i.e., Harbin, Beijing, Wuhan, Chongqing, Hangzhou, and Guangzhou. Daily solar radiation data from 1991 to 2020 in these locations were believed to be reliable after strict data quality control made by NMIC, so the datasets from 1991 to 2010 were used for model calibration, while the datasets from 2011 to 2020 were used for model validation in this study.

### 2.2. Calculation of UTCI

UTCI is defined as the isothermal air temperature which would elicit the same response under a set of reference conditions [7]. UTCI is calculated by solving Fiala’s heat balance model [3], which calculates the human physical response to meteorological conditions. The model is based on a thermoregulation model, consisting of 12 human body elements and 187 tissue nodes [3]. A rapid calculation of UTCI can be achieved by a polynomial approximation procedure to compute the offset of UTCI to *T_a_* (UTCI−*T_a_*) as follows [16].

(1) $$\;\mathrm{UTCI}-T_{a}=f\left(T_{a},V,e,T_{mrt}-T_{a}\right)$$

where *T_a_* denotes the air temperature, *V* the wind speed, *e* the vapor pressure, and *T_mrt_* the mean radiative temperature, respectively. *V*, *e*, and *T_mrt_* can be computed as:

(2) $$\;V=\sqrt{V_{u}^{2}+V_{v}^{2}}\;\;\;$$

(3) $$e=6.11\exp\left[5417.753\left(\frac{1}{273.16}-\frac{1}{273.16+T_{d}}\right)\right]$$

(4) $$\;\;\;\;\;\;\;\;\;\;\;T_{mrt}={\left(\frac{R_{p}+0.5L_{g}+0.5L_{a}}{{0.95\times 5.667\times 10}^{-8}}\right)}^{1/4}-273$$

(5) $$L_{g}={5.5\times 10}^{-8}{\times (273+T_{g})}^{4}$$

(6) $$L_{a}={5.5\times 10}^{-8}{\times (273+T_{a})}^{4}\times (0.82-{0.25\times 10}^{-0.094\times e})$$

where *V_u_* and *V_v_* are wind speed at meridional and longitudinal directions, respectively; *T_d_* is the dew point temperature, *T_g_* is the ground temperature, and *R_p_* is the solar radiation observed by a nude man, which can be estimated by the SolAlt model [9,44].

UTCI is divided into 10 categories ranging from extreme cold stress to extreme heat stress [16] (Table 2). This category has been extensively validated by both climate chamber and wind tunnel experiments [3,19], and widely accepted by both the International Society on Biometeorology [3,7,16] and the researchers in this domain [21,22,23,24,25,26]. Currently, calculation of UTCI can be performed by Bioklima 2.6 software package with four meteorological input variables, including solar radiation, air temperature, vapor pressure or humidity, and wind speed [9,23]. Detailed description on the input variables can be found in the relevant procedures and processing steps [44].

### 2.3. Estimation of Solar Radiation

Two sunshine- and two temperature-based empirical models, together with two machine-learning methods, were used to estimate solar radiation in this study.

#### 2.3.1. Angstrom Model

The Angstrom formula is the most widely used popular empirical sunshine-based model [46,47], which calculates solar radiation with sunshine hours as follows.

(7) $$R_{a}=R_{e}\times (a+b\frac{S}{S_{0}})$$

where *R_a_* is the observed daily solar radiation, *R_e_* the extra-terrestrial solar radiation, *S* the observed sunshine hours, and *S*_0_ the potential sunshine hours, respectively. *R_e_* and *S*_0_ can be calculated with the method recommended by FAO [48].

#### 2.3.2. Ogelman Model

The Ogelman model is also an empirical sunshine-based model, which can be expressed as [49]

(8) $$R_{a}=R_{e}\times [a+b\frac{S}{S_{0}}+c{\left(\frac{S}{S_{0}}\right)}^{2}]$$

#### 2.3.3. Bristow Model

The Bristow model is an empirical temperature-based model, using temperature as input variables to predict solar radiation [50].

(9) $$\;\;R_{a}=a\times R_{e}\times [1-\exp\left(-b\times T^{c}\right)]$$

where ΔT is the difference between daily maximum and minimum temperature.

#### 2.3.4. Hargreaves Model

The Hargreaves model is also an empirical temperature-based model, estimating solar radiation with the diurnal range of temperature [51].

(10) $$R_{a}=R_{e}\times \left(a\times \Delta T^{0.5}+b\right)$$

#### 2.3.5. BP Neural Network

BP neural network has one or more hidden layers, and one output layer. The data are propagated from input layer to output layer through hidden layer with error being transmitted in the opposite direction, so the connection weight of the network can be corrected to decrease the final error. Recently, BP neural has been argued to be efficient in solar radiation estimation [52].

#### 2.3.6. Support Vector Machine

The support vector machine (SVM) is a supervised machine-learning method for data analysis and pattern recognition. The SVM follows the concept of separating the features from one another. According to the algorithm of SVM, the same types of features are set on one plane. To be specific, the SVM aims to find a hyperplane that can separate data points of one class from another to the best degree. The best degree is referred to as the hyperplane with the largest margin between the two classes, and margin is defined as the biggest width of the slab parallel to the hyperplane that has no interior data points. Based on the principle of structural risk minimization, this method can better solve the problems with nonlinearity and high dimensionality. Up to now, SVM has been widely employed for radiation estimation due to its high accuracy [41].

### 2.4. Statistical Analysis

The Nash–Sutcliffe Efficiency (*NSE*), the Mean Absolute Percentage Error (*MAPE*), and the Root Mean Square Error (*RMSE*) were used as criteria to evaluate the model performance [39,53]. *NSE* is analogous to coefficient of determination, with the exception that NSE ranges from negative infinity to 1, which can be used for indicating model efficiency. The negative value of *NSE* indicates that the mean observation can be used as a better predictor than the simulated values. *MAPE* is used to identify the relative bias in simulated values compared with observations, while *RMSE* is an indicator of the squared difference between simulated and measured values.

(11) $$\;NSE=1-\frac{\sum_{i=1}^{n}{\left(O_{i}-S_{i}\right)}^{2}}{\sum_{i=1}^{n}{\left(O_{i}-\bar{O}\right)}^{2}}$$

(12) $$MAPE=\frac{\sum_{i=1}^{n}\left|\frac{O_{i}-S_{i}}{O_{i}}\right|\times 100}{n}$$

(13) $$\;RMSE={\left[\frac{1}{n}\overset{n}{\underset{i=1}{\sum }}{\left(O_{i}-S_{i}\right)}^{2}\right]}^{\frac{1}{2}}$$

where *O_i_* is the observed value, *S_i_* the estimated value, $\bar{O}$ the average of the observed value, and *n* the sample number of observations, respectively. Higher *NSE* and lower *MAPE* and *RMSE* mean better model performance.

Trends in time series of UTCI were estimated by the nonparametric Theil–Sen’s estimator [54].

(14) $$\beta =Median\left(\frac{X_{j}-X_{i}}{j-i}\right),\;i<j\;$$

where *X_i_* and *X_j_* are the UTCI values for year *i* and *j*, respectively. Positive *β* denotes an increase in trend, while negative value of *β* indicates decrease in the time series. The trend significance was tested by Mann–Kendall method. Detailed information on calculating the standardized test statistic (*z*) of MK can be referred to the relevant descriptions [10,55,56].

The empirical coefficients of *a*, *b* and *c* in Equations (7)–(10) were fitted by numerical iteration methods [53]. The BP and SVM methods were implemented through the “nnet” and “e1071” packages in R language, respectively. A regression task was involved in the study, and both the empirical models and the machine-learning methods were calibrated and validated before application. The machine-learning methods are believed to be more accurate in radiation estimation than the empirical models [41], but they require big data for model training. In contrast, the empirical models are easy to operate, and require less data for model calibration than the machine-learning methods.

## 3. Results

### 3.1. Comparison of Model Performance in Radiation Estimation and UTCI Calculation

Coefficients of the empirical models were calibrated with the dataset from 1991 to 2010, and the calibration results are shown in Table 3. For the sunshine-based Angstrom model, the coefficient *a* ranged from 0.130 to 0.240 with averaged value of 0.16, while the coefficient *b* ranged from 0.456 to 0.587 with an average of 0.528. The values of *NSE* were between 0.861 and 0.930 with an average of 0.886, indicating that Angstrom had a high efficiency in radiation estimation. The average *MAPE* and *RMSE* were 16.795 and 27.452, respectively. The calibration performance of the Ogelman model was very similar to that of the Angstrom model. The average *NSE* was 0.895 for the Ogelman model, which was almost equal to that of the Angstrom model. In addition, the values of *MAPE* and *RMSE* were also very close to those of the Angstrom model, either for each location or as a whole. For temperature-based models, the average value of *NSE* was 0.673 for the Bristow model, and the average values of *MAPE* and *RMSE* were 22.94 and 46.386, respectively. The Hargreaves model showed a similar performance as the Bristow model with very close *NSE*, *MAPE*, and *RMSE* values.

**Figure 2:**
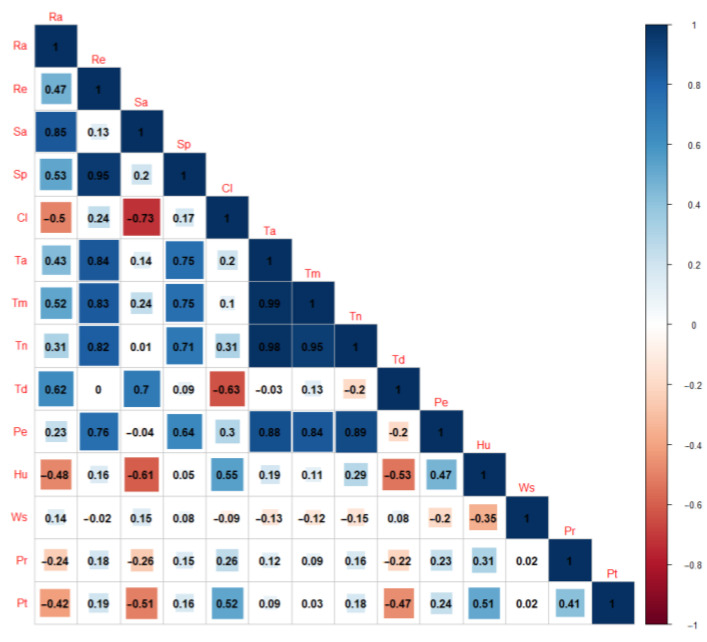
Correlations among different meteorological items.

Correlation analysis identified that many meteorological factors should be used as input variables for training machine leaning methods, including extra-terrestrial solar radiation (*R_e_*), sunshine hours (*S_a_*), potential sunshine hours (*S_p_*), cloudiness (*Cl*), air mean temperature (*T_a_*), maximum temperature (*T_m_*), minimum temperature (*T_n_*), diurnal variation of temperature (*T_d_*), vapor pressure (*P_e_*), humidity (*H_u_*), wind seep (*W_s_*), precipitation (*P_r_*), and precipitation events (*P_t_*) (Figure 2). However, the machine-learning models could be over-trained when the highly correlated features were used as input variables simultaneously. In this study, when the correlation coefficients among several features were higher than 0.8, only one feature was used as the input variable for the machine-learning models. According to this rule, together with the consideration of the data availability, *T_a_* was kept as input variable for machine-learning models, while *T_m_*, *T_n_*, and *P_e_* were removed from the dataset. However, all of the three sunshine-related features, including *S_p_*, *R_e_*, and *S_a_*, were used as input variables despite of their high correlation coefficients, as removal of any one of these features would lead to poor performance of the machine-learning models. The machine-learning models were also trained with the dataset from 1991 to 2011 for comparison with the results by the empirical models (Table 3). A “Trial and error” method was used to tune the machine-learning models to determine the best parameters. For the BP model, the number of units in the hidden layer and the parameter for weight decay were set as 10 and 0.01, respectively. For the SVM model, the kernel used in training and predicting was set as “radial” basis, and the cost of constraints violation was tuned as 1. The value of gamma was determined as 0.125. The two machine-learning methods performed better in calibration with higher *NSE* and lower *MAPE* and *RMSE*. The average values of *NSE*, *MAPE*, and *RMSE* were 0.938, 14.210, and 20.127, respectively, for the BP neural network, while the Support Vector Machine further improved the model performance in calibration by a slightly higher *NSE* of 0.940, and lower *MAPE* and *RMSE* of 13.711 and 19.781, respectively.

The calibrated empirical models and trained machine-learning models were used for validation against the observed radiation from 2011 to 2020, respectively. The validation results are shown in Table 4. For each empirical model, the overall model performance in validation was quite similar to that in calibration. However, compared to the performance in calibration, the machine-learning methods showed worse performance in validation. The average value of *NSE* was 0.878 for the BP neural network, much lower than that of 0.938 in calibration. In addition, the values of *MAPE* and *RMSE* also became larger in model validation for machine-learning methods.

**Figure 3:**
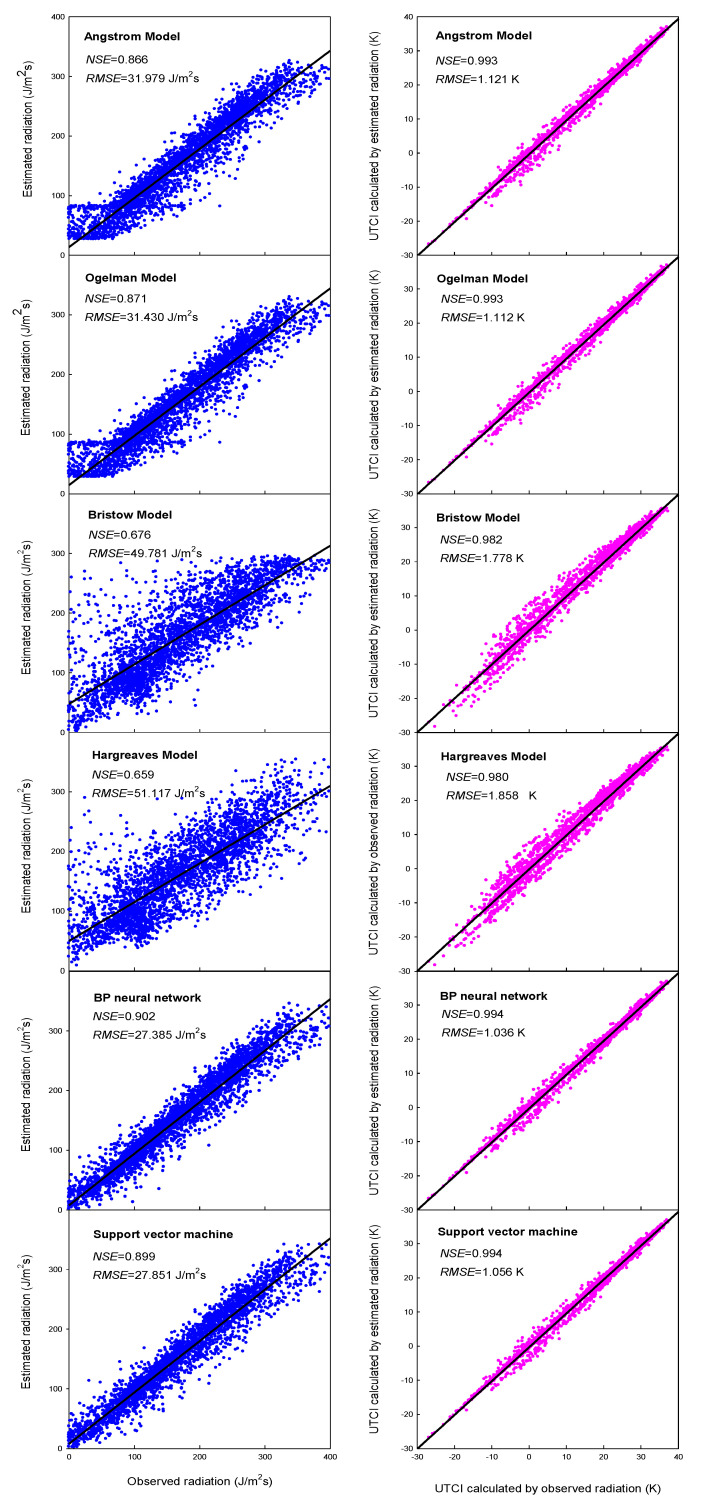
Validation of the radiation estimation and UTCI calculation in Beijing by different methods.

**Figure 4:**
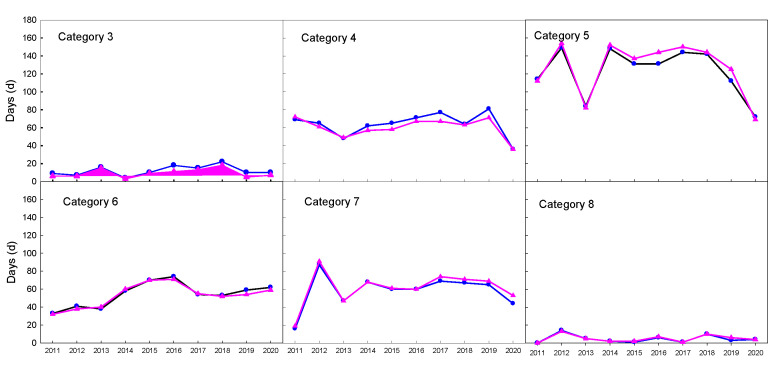
Comparison of the days within each UTCI category calculated by observed radiation (line blue circle) with those by estimated radiation (line with pink triangle).

The estimated radiation dataset in 2011–2020 was used to calculate UTCI, and the obtained UTCI was compared to that calculated with observed radiation. The UTCI validation result is shown in Table 5. As a whole, the error in radiation estimation was not amplified, but reduced in the UTCI calculation process. The sunshine-based Angstrom model had a high average *NSE* value of 0.990, and lower *RMSE* value of 1.236 (Table 5). Referred to the validation in radiation (Table 4), an average error of 35.4 J/m^2^ s radiation estimation led to an average error of 1.2 °C in UTCI calculation, which was exactly within the error range of 2.1 °C identified by sensitivity analysis [4]. The Ogelman model showed very similar performance to the Angstrom model. However, the temperature-based models, both the Bristow and Hargreaves models, presented lower *NSE* and higher *MAPE* and *RMSE* than sunshine-based models. In contrast, the *NSE* values of the machine-learning methods were 0.992, slightly higher than those of the sunshine-based models. The average *RMSE* value for both machine-learning methods was about 1.1 °C, showing very limited advantage over the sunshine-based models. Considering both accuracy and applicability, the Angstrom model was selected to estimate solar radiation for UTCI calculation in regional analysis below, due to its easy calibration and readily available input data. The accuracy of the Angstrom model in calculating UTCI and day number within each category can be seen in Figure 3 and Figure 4, respectively.

### 3.2. Spatial Analysis of UTCI and Day Number within Each Category

**Figure 5:**
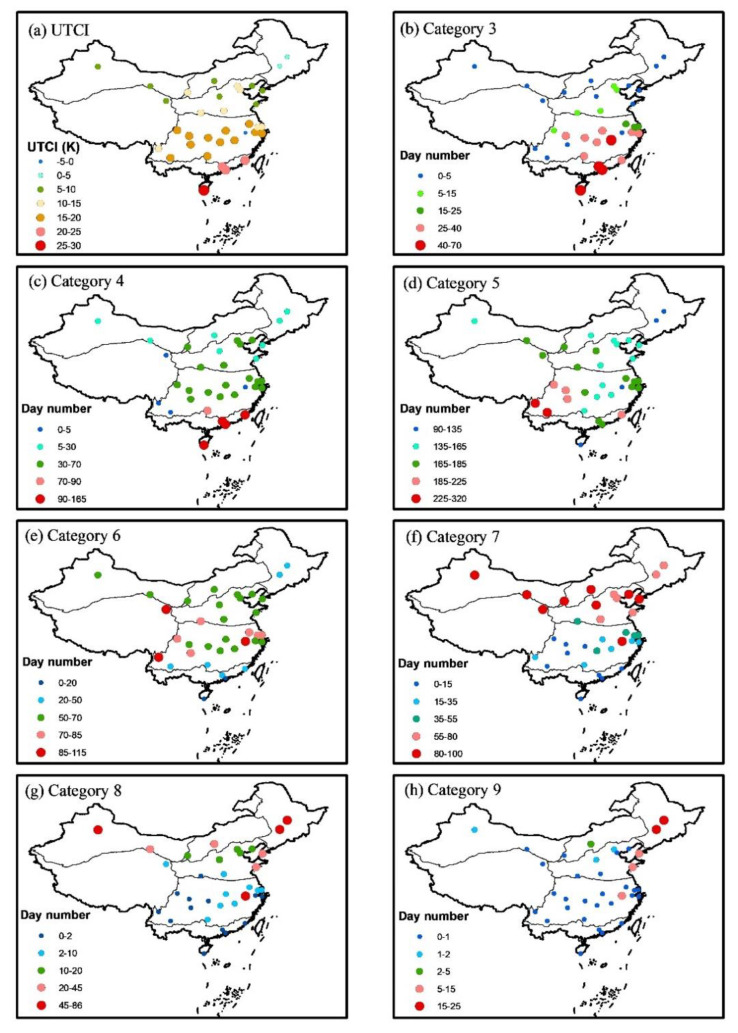
Spatial distribution of UTCI and yearly days within each category in the large tourism cities of China.

The calibrated model was used to calculate the UTCI in all of the tourism cities from 1961 to 2020. The spatial distribution of average yearly UTCI and the days within each category in Chinese tourism cities are shown in Figure 5a, and the detailed information is shown in Table 6. As a whole, the UTCI increased gradually from north to south in the tourism cities of China, with the exception of Huangshan location due to its high altitude (see Table 1). UTCI in the tourism cities of northeast China was lower than 5 °C, while the UTCI reached the highest value around 28 °C in Sanya, the southernmost part of China. The days within each category are shown in Figure 5b–h. For the days under no thermal stress (Figure 5d), Kunming and Lijiang had the largest value between 225 and 320 days. The tourism cities in northeast and southeast China had the lowest values between 90 and 135 days with the lowest and highest UTCI, respectively. The tourism cities in the most developed regions such as the Yangtze River Delta and Zhujiang River Delta had about 170 days under no thermal stress in a year. The distribution of days under slightly cold stress was very similar to that under no thermal stress (Figure 5e).

In contrast with the spatial distribution of days under no thermal stress and slightly cold stress, more days under heat stress were identified in the tourism cities of south China (Figure 5b,c), while more days under cold stress were found in the tourism cities of north China (Figure 5f–h). According to category 3, many tourism cities in the lower reach of Yangtze River, including Wuhan, Nanjing, Hangzhou, and Shanghai etc., had about one month under strong heat stress, while the tourism cities in Zhujiang River Delta such as Guangzhou and Shenzhen had about two months under strong heat stress in a year. Sanya, the southernmost China, experienced 70 days under strong heat stress in a year. 

Strong cold stress mainly occurred in the tourism cities of north China. The largest days under strong cold stress occurred in Harbin and Changchun in northeast China and Wulumuqi in northwest China, in a range from 45 to 86 days in a year (Figure 5g). Days under very strong cold stress occurred very seldom in most tourism cities of China. Many locations, 28 out of 35 cities, had less than two days under very strong cold stress, most of which were located in south China (Figure 5h). However, Changchun and Harbin in northeast China did experience very strong cold stress in a year with day number between 15 and 25. 

### 3.3. Temporal Trend in UTCI and Day Number within Each Category

**Figure 6:**
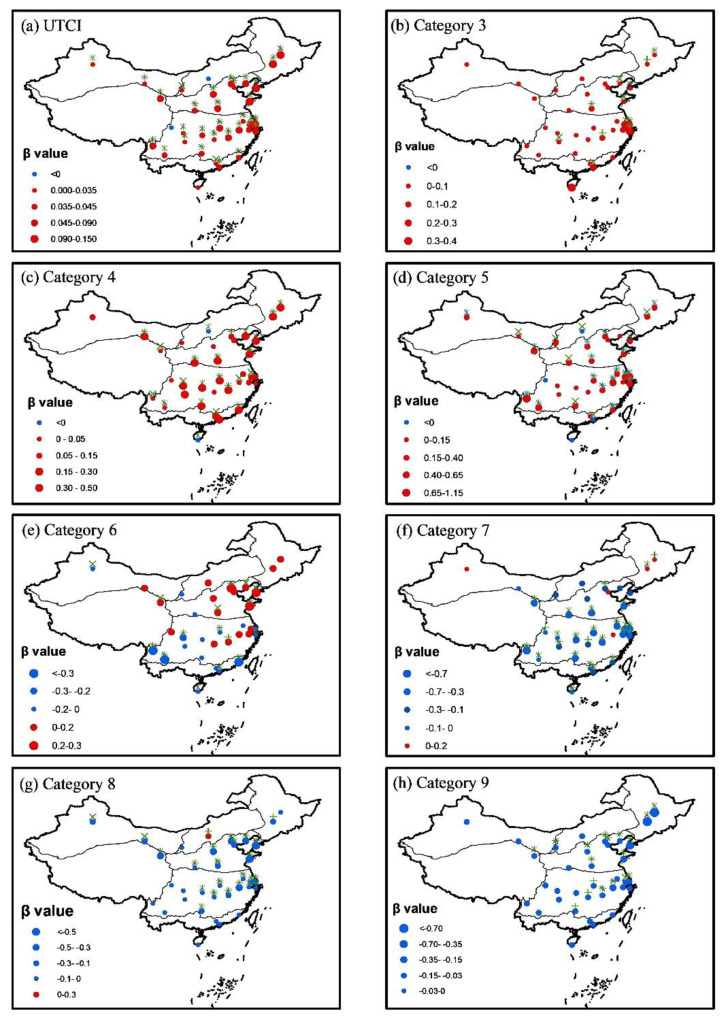
Trend analysis of the changes in yearly day number within each category from 1961 to 2020. Plus +, multiple ×, and asterisk * signs denote confidence levels of 90%, 95%, and 99%, respectively.

The time analysis was further conducted with the calculated UTCI in all of the tourism cities from 1961 to 2020, and the trend analysis of the UTCI and days number within each category from 1961 to 2020 is presented in Figure 6. On the whole, the UTCI showed an overall increasing trend for most of the large tourism cities in China (Figure 6a), most of which increased with very high statistical significance level of 99% (*z* > 2.58). Only 2 out of 35 tourism cities showed negative trends in UTCI. Huhehaote had a negative trend with very small value of −0.009 °C/a, and the negative trend in UTCI was negligible in Chengdu with an even smaller value of −0.001 °C/a. In addition, both trends did not pass the significant level of 90% (*z* < 1.96). In other words, very slight decreases in both locations were not significant in terms of statistical analysis. Generally speaking, the days under no thermal stress increased in most of the tourism cities over China (Figure 6d). The days under slight cold stress increased in most locations in the northern parts of China, while they decreased in most locations in the southern parts of China (Figure 6e). The days under moderate heat stress and strong heat stress generally increased over China from 1961 to 2000 (Figure 6b,c). In contrast with these increasing trends, trends in the days under moderate cold, strong cold, and very strong cold stresses presented an overall decreasing trend in most locations over China (Figure 6f–h). Especially, the days under very strong cold stress decreased in all locations of China (Figure 6h). In short, the UTCI increased in most locations of China, accompanying with an overall increase in days under no thermal stress and heat stress, and an overall decrease in days under cold stress (Figure 6a–h).

## 4. Discussion

### 4.1. Optimal Strategy on Estimating Solar Radiation for UTCI Calculation

The sunshine-based Angstrom model was selected as the best choice to estimate solar radiation for UTCI calculation. In this study, the sunshine-based models performed better than the temperature-based models in terms of higher *NSE* and lower *MAPE* and *RMSE* (Table 3 and Table 4), which was in agreement with the results from the previous reports [33,38]. The machine-learning method is believed to be a promising choice for accurate estimation of solar radiation [41], but the strict requirement of many input variables for model training cannot always be met, which inevitably leads to better performance in calibration but worse performance in validation [57]. This inherent defect was again identified by the higher *NSE* in calibration and the lower *NSE* in validation in this study (Table 3 and Table 4). In fact, the machine-learning methods can only slightly improve the accuracy in radiation estimation compared with the sunshine-based models, but require many more input variables and datasets for model training [41]. Due to limitation of length, only the BP and SVM methods were implemented in this study. Recently, the Random Forest method has also been identified as an effective algorithm in radiation estimation [58], which may outperform the BP and SVM methods. So, involvement of more machine-learning methods to further improve accuracy in radiation estimation becomes necessary in the future research work.

Based on sensitivity analysis, Weihs et al. [4] concluded that the maximum uncertainty in solar radiation estimation would be 15% in conventional sites, which might contribute to a maximum uncertainty in UTCI with 2.1 °C. In this study, the RMSE of the radiation by the Angstrom model was 32 J/m^2^ s in Beijing, while the average solar radiation was around 300 J/m^2^ s in clear days (Figure 3). This error resulted in an uncertainty of about 11% in radiation estimation, which further led to an uncertainty of about 1.2 °C in UTCI calculation (Figure 3), just within the error range made by sensitivity analysis [4]. Therefore, it can be reasonably concluded that accurate UTCI can be obtained through the optimal strategy for radiation estimation. Based on the results and analysis above, the sunshine-based Angstrom model is recommended as the optimal strategy due to its excellent model performance, easy operation, and readily available input variables.

### 4.2. Increase in Yearly Day Number under no Thermal Stress Accompanying with More Risks in Heat Stress in Summer in China

**Figure 7:**
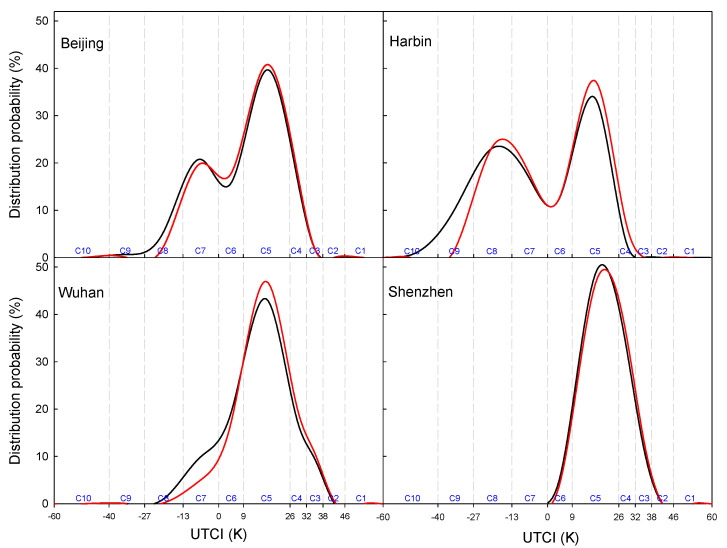
Distribution probability of the days within each category in the period of 1961–1990 (black lines) and 1991–2020 (red lines).

Global warming has been well acknowledged in modern society [59], with its great negative effects in many aspects such as the economy [60], agriculture [61], and global food security [62]. In addition, climate change is also believed to give rise to more extreme weather events, which would pose great risks to the public health [63,64,65,66,67]. Considering so many impressive negative impacts, people would naturally envisage that days under no thermal stress would decrease in the context of climate change. However, this study showed an opposite result in that the yearly day number under no thermal stress increased in most tourism cities over China in the recent six decades (Figure 6d). To investigate its reliability, the probability of days under each category in the period of 1961–1990 and 1991–2020 was analyzed, respectively. Figure 7 clearly indicates that the distribution probability curve slightly right-shifted in the tourism cities of China. This shift can be attributed to the decrease in cold days and increase in warm days caused by climate warming. For most locations, more days under cold stress changed to the days under no thermal stress, while fewer days under no thermal stress shifted to the days under heat stress. This might be the exact reason for the increasing days under no thermal stress in most tourism cities of China. Only for very few tourism cities such as Shenzhen was this shift was not significant due to the high temperature in south part of the tropical humid zone. In short, it is the asymmetric changes in the hot days and cold days that led to the increase in days under no thermal stress within a year in the tourism cities of China. This argument is strongly justified by the previous report given by Zhai et al. [68], who identified that the number of hot days displayed a slight increasing trend, while the number of the frost days exhibited a significant decreasing trend in China in the recent decades.

**Figure 8:**
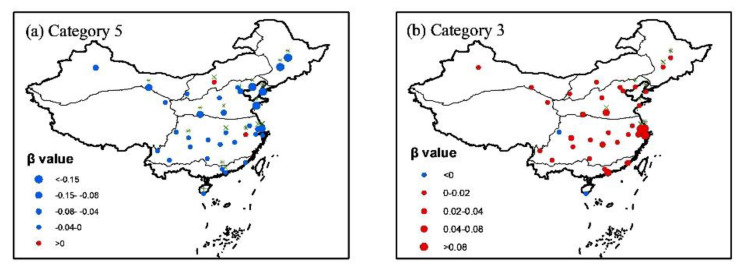
Trend analysis of the changes in day number under no thermal tress (**a**) and strong heat stress (**b**) in summer from 1961 to 2020.

The increase in days under no thermal stress mainly occurred in the spring and autumn. In summer, the days under no thermal stress did decease (Figure 8a). Without consideration of the radiation effect on UTCI, Yan et al. [10] also believed that days under no thermal stress have been decreasing in most locations of China from 1961 to 2016 in summer. In contrast to the decrease in days under no thermal stress, the days under strong heat stress showed an increasing trend in most tourism cities in summer (Figure 8b), which would inevitably cause more potential heat threats to the public health and make the further investigation on thermal perception essential and urgent [28].

### 4.3. Certainties and Uncertainties in UTCI Estimation over China

It is certain that UTCI deserves its name, as the term “universal” means that UTCI is appropriate for all assessments of the outdoor thermal conditions in the major human bio-meteorological applications [7]. Previously, many simple thermal indices have already been suggested in different regions in China [69,70,71], but none of them could be used for the spatial analysis at the national scale over China due to regional adaptability. In contrast, UTCI successfully revealed the overall distribution of the thermal environments in the tourism cities of China (Figure 5), and the results are highly consistent with public cognition. For example, Kunming is called “spring city” due to its long period of thermal comfort, which has been identified by UTCI as the city with the most days under no thermal stress (Figure 5d). The general distribution of more cold stress in tourism cities of north China and more heat stress in tourism cities of south China also agree well with the basic public cognition. However, it should be noted that Huangshan is an exception of this the general spatial distribution of UTCI (Figure 5a). Though located in the central part of China, the yearly UTCI of Huangshan is much lower than the surrounding cities due to its high altitude (Table 1). This unique UTCI makes Huangshan become one of the most attractive tourism destinations in the summertime. So, the effect of topographic features on UTCI should be explored in the future research relevant to the distribution of thermal environments in China. 

The general spatial distribution of UTCI in the tourism cities of China is comparable to the previous reports [9,44], with quantitative differences due to different strategies on dealing with input variables. In the future, error in UTCI calculation caused by using cloudiness [9] or simple interpolation [44] should be examined deliberately. In addition, UTCI stress should be redefined at daily scale. Currently, UTCI category is defined at hourly scale [16]. However, air temperature has obvious seasonal and daily changes, especially in the monsoon climate regions such as China [72]. As air temperature is the dominant element of UTCI, and UTCI also has seasonal and daily changes at daily and seasonal scales. Therefore, both heat stress and cold stress would be underestimated if the daily UTCI were classified according to the current UTCI category at the hourly scale. For example, the daily UTCI of 26 °C was defined as no thermal stress (9–26 °C) according to the current hourly category (Table 2). However, there is a great possibility that UTCI is higher than 26 °C in many hours during the day, so it actually should be classified as moderate heat stress rather than no thermal stress in this day. Vice versa, the daily UTCI of 9 °C should actually be referred to as a slight cold day, as UTCI would be lower than 9 °C in many hours of the day. Likewise, daily UTCI values within category 3 or category 9 might actually belong to category 2 or category 10. This might be the exact reason that no very strong heat stress (category 10) and extreme hot stress (category 2) were identified in both this study and all of the previous reports, which is obviously contradicted to the public cognition that there are many “ice” and “furnace” cities in China. Thus, it is urgent to redefine UTCI category at daily scale through comparison of daily UTCI with hourly values in the future work.

## 5. Conclusions

The sunshine-based Angstrom model performed well in estimation of solar radiation in the tourism cities of China with high *NSE* of 0.864 and low *RMSE* of 35.4, which resulted in a high efficiency in UTCI calculation with *NSE* of 0.99. Uncertainty in UTCI calculation with estimated solar radiation by the Angstrom model was 1.2 °C, just within the error range obtained by sensitivity analysis. So, the Angstrom model is proposed as the optimal strategy on solar radiation estimation for UTCI calculation due to its high accuracy and easy operation.

Spatial distribution of UTCI indicated that day number under no thermal stress was higher in the tourism cities of central China, within a range from 135 to 225 days, and the largest days under no thermal stress occurred in Kunming and Lijiang in southwest China. Very strong cold stress mainly occurred in Harbin and Changchun in northeast China, with a period between 15 and 25 days in a year. Strong heat stress mainly occurred in the tourism cities of south China, especially southernmost Sayan, with a period between 40 and 70 days in a year.

Contrary to popular belief, days under no thermal stress during a year have increased in most tourism cities of China in the recent decades in the context of global warming, which can be attributed to the asymmetric changes in the significant decrease in frost days and slightly increase in hot days over China in the recent decades. However, in the summer, days under no thermal stress have decreased in most regions of China, accompanying with increasing trends in days under very strong heat stress, especially in the developed regions such as Yangtze River Delta and Zhujiang River Delta, which would pose great risks to thermal perception for the public health in these regions.

UTCI can successfully identify the general spatial distribution and temporal trend of thermal environments in most tourism cities of China. However, up to now, all reports on Chinese UTCI were performed by classifying daily UTCI values according to the category obtained at hourly scales, which would result in the underestimation of days under extreme hot or cold stress conditions. This is exactly the weakness of UTCI performance in China, i.e., no extreme cold or extreme hot days have been identified by the daily UTCI values. Thus, it is urgent to redefine UTCI category at daily scales by comparing daily UTCI with the corresponding hourly values in the future research work.

## Figures and Tables

**Table 1:** Detailed information of the tourism cities in China.

Cities	Latitude (N)	Longitude (E)	Altitude (m)	Climate Conditions	Population (Million)
Beijing (BJ)	39.8	116.5	31.3	temperate and sub-humid	21.9
Tianjin (TJ)	39.1	117.1	3.5	temperate and sub-humid	13.9
Dalian (DL)	38.9	121.6	91.5	temperate and sub-humid	7.5
Qingdao (QD)	36.1	120.3	76.0	temperate and sub-humid	10.0
Shanghai (SH)	31.4	121.5	5.5	subtropical humid	24.9
Nanjing (NJ)	31.9	118.9	35.2	subtropical humid	9.3
Suzhou (SZ)	31.3	120.6	10.7	subtropical humid	12.7
Hangzhou (HZ)	30.2	120.2	41.7	subtropical humid	12.2
Xiamen (XM)	24.5	118.1	139.4	tropical humid	5.2
Guangzhou (GZ)	23.2	113.3	41.0	tropical humid	18.7
Shenzhen (SE)	22.5	114.0	63.0	tropical humid	17.6
Sanya (SY)	18.2	109.6	419.4	tropical humid	1.0
Qinhuangdao (QH)	39.9	119.5	2.4	temperate and sub-humid	3.1
Ningbo (NB)	30.0	121.6	4.0	subtropical humid	8.5
Harbin (HB)	45.8	126.8	142.3	temperate and sub-humid	10.0
Zhengzhou (ZZ)	34.7	113.7	110.4	temperate and sub-humid	12.6
Wuhan (WH)	30.6	114.1	23.6	subtropical humid	12.3
Zhangjiajie (ZJ)	29.1	110.5	183.5	subtropical humid	1.5
Changsha (CS)	28.2	112.9	68.0	subtropical humid	10.0
Huangshan (HS)	30.1	118.2	1840.4	subtropical humid	1.3
Guilin (GL)	25.3	110.3	164.4	subtropical humid	4.9
Changchun (CC)	43.9	125.2	236.8	temperate and sub-humid	9.1
Hohhot (HH)	40.8	111.7	1063.0	Inner Mongolia	3.4
Jinzhong (JZ)	37.7	112.8	831.2	Temperate and sub-humid	3.3
Nanchang (NC)	28.6	115.9	46.9	subtropical humid	6.4
Xi’an (XA)	34.3	108.9	397.5	temperate and sub-humid	12.9
Chongqing (CQ)	29.5	106.5	351.1	subtropical humid	32.1
Chengdu (CD)	30.7	104.0	507.3	subtropical humid	21.2
Kunming (KM)	25.0	102.7	1888.1	subtropical humid	8.5
Lijiang (LJ)	26.9	100.2	2380.9	subtropical humid	1.3
Zunyi (ZY)	27.7	106.9	843.9	subtropical humid	6.6
Yinchuan (YC)	38.5	106.2	1110.9	Inner Mongolia	2.9
Jiuquan (JQ)	39.8	98.5	1477.2	temperate and warm-temperate	1.0
Xining (XN)	36.7	101.8	2295.2	Qinghai-Tibetan Plateau	2.5
Wulumuqi (WL)	43.8	87.7	935.0	temperate and warm-temperate	4.1

**Table 2:** Category for UTCI in terms of thermal stress (according to Brode et al. [16]).

Category	UTCI Range (°C)	Stress Description
Category 1 (C1)	above +46	extreme heat stress
Category 2 (C2)	+38 to +46	very strong heat stress
Category 3 (C3)	+32 to +38	strong heat stress
Category 4 (C4)	+26 to +32	moderate heat stress
Category 5 (C5)	+9 to +26	no thermal stress
Category 6 (C6)	0 to +9	slight cold stress
Category 7 (C7)	−13 to 0	moderate cold stress
Category 8 (C8)	−27 to −13	strong cold stress
Category 9 (C9)	−40 to −27	very strong cold stress
Category 10 (C10)	below −40	extreme cold stress

**Table 3:** Calibration results of the empirical models and machine-learning methods on estimation of the solar radiation.

Model	City	*a*	*b*	*c*	*NSE*	*MAPE*	*RMSE*	*Slope*	*Inter*	*n*
Angstrom	Beijing	0.167	0.518	-	0.930	11.677	21.408	0.894	15.637	7118
	Hangzhou	0.136	0.587	-	0.900	17.061	27.982	0.881	15.355	6993
	Guangzhou	0.159	0.502	-	0.844	16.697	26.249	0.840	21.090	6665
	Harbin	0.240	0.456	-	0.900	13.338	27.154	0.880	18.462	7006
	Wuhan	0.130	0.537	-	0.881	17.865	29.602	0.867	17.049	7011
	Chongqing	0.133	0.570	-	0.861	24.131	30.517	0.858	13.370	7020
	Average	0.161	0.528	-	0.886	16.795	27.152	0.870	16.827	6969
Ogelman	Beijing	0.174	0.483	0.038	0.930	11.501	21.422	0.895	16.510	7118
	Hangzhou	0.122	0.832	−0.310	0.908	17.158	26.837	0.890	14.371	6993
	Guangzhou	0.141	0.743	−0.303	0.858	16.700	25.032	0.873	17.165	6665
	Harbin	0.224	0.585	−0.138	0.902	13.574	26.910	0.882	18.270	7006
	Wuhan	0.116	0.793	−0.316	0.890	18.152	28.481	0.874	16.323	7011
	Chongqing	0.123	0.906	−0.481	0.879	24.012	28.547	0.868	12.635	7020
	Average	0.150	0.724	−0.252	0.895	16.850	26.205	0.880	15.879	6969
Bristow	Beijing	0.602	0.025	1.840	0.670	20.070	46.432	0.711	47.635	7118
	Hangzhou	0.549	0.009	2.428	0.691	23.948	49.186	0.695	41.642	6993
	Guangzhou	0.515	0.011	2.336	0.600	23.024	42.090	0.625	47.369	6663
	Harbin	0.600	0.081	1.324	0.699	21.321	47.050	0.685	48.281	7006
	Wuhan	0.496	0.005	2.938	0.626	24.209	52.551	0.607	53.517	7011
	Chongqing	0.563	0.015	1.978	0.750	25.154	41.006	0.753	24.299	7020
	Average	0.554	0.024	2.141	0.673	22.954	46.386	0.679	43.791	6969
Hargreaves	Beijing	0.180	−0.098	-	0.649	21.504	47.874	0.688	51.044	7118
	Hangzhou	0.244	−0.304	-	0.693	24.305	49.000	0.678	44.199	6993
	Guangzhou	0.248	−0.325	-	0.604	22.791	41.858	0.597	53.491	6663
	Harbin	0.137	0.046	-	0.698	21.220	47.096	0.695	47.187	7006
	Wuhan	0.242	−0.296	-	0.625	23.737	52.674	0.572	55.445	7011
	Chongqing	0.220	−0.288	-	0.770	23.042	39.282	0.748	25.765	7020
	Average	0.212	−0.211	-	0.673	22.767	46.297	0.663	46.189	6969
BP neural network	Beijing	-	-	-	0.960	9.745	16.197	0.958	6.336	7118
	Hangzhou	-	-	-	0.947	14.718	20.307	0.947	7.359	6993
	Guangzhou	-	-	-	0.925	13.429	18.252	0.922	10.088	6665
	Harbin	-	-	-	0.925	11.900	23.519	0.922	11.988	7006
	Wuhan	-	-	-	0.917	17.318	24.701	0.917	11.456	7011
	Chongqing	-	-	-	0.951	18.147	18.083	0.951	4.857	7020
	Average	-	-	-	0.938	14.210	20.177	0.936	8.681	6969
Support vector machine	Beijing	-	-	-	0.960	9.777	16.155	0.965	5.478	7118
	Hangzhou	-	-	-	0.947	14.451	20.369	0.942	7.554	6993
	Guangzhou	-	-	-	0.925	13.183	18.174	0.927	10.171	6665
	Harbin	-	-	-	0.927	11.256	23.241	0.915	11.244	7006
	Wuhan	-	-	-	0.929	16.153	22.959	0.950	7.703	7011
	Chongqing	-	-	-	0.953	17.445	17.786	0.952	4.932	7020
	Average	-	-	-	0.940	13.711	19.781	0.942	7.847	6969

**Table 4:** Validation results of the empirical models and machine-learning methods on estimating solar radiation.

Model	City	*NSE*	*MAPE*	*RMSE*	*Slope*	*Inter*	*n*
Angstrom	Beijing	0.866	14.284	31.979	0.823	13.784	3157
	Hangzhou	0.839	21.244	39.101	0.794	14.421	3181
	Guangzhou	0.848	18.853	28.226	0.849	8.514	3048
	Harbin	0.758	17.935	42.942	0.745	30.079	2982
	Wuhan	0.838	21.756	35.622	0.838	6.737	3090
	Chongqing	0.865	25.230	34.305	0.833	11.752	2666
	Average	0.836	19.884	35.363	0.814	14.215	3021
Ogelman	Beijing	0.871	13.925	31.430	0.824	14.753	3157
	Hangzhou	0.850	20.544	37.641	0.805	13.005	3181
	Guangzhou	0.859	18.600	27.114	0.870	5.298	3048
	Harbin	0.766	17.675	42.292	0.758	28.327	2982
	Wuhan	0.851	20.876	34.192	0.851	5.718	3090
	Chongqing	0.869	25.018	33.765	0.812	13.284	2666
	Average	0.844	19.440	34.406	0.820	13.398	3021
Bristow	Beijing	0.676	20.839	49.781	0.663	47.803	3156
	Hangzhou	0.688	27.359	54.323	0.665	36.553	3180
	Guangzhou	0.673	22.910	41.338	0.670	39.519	3047
	Harbin	0.616	23.049	54.105	0.602	55.703	2981
	Wuhan	0.631	25.879	53.798	0.612	58.487	3088
	Chongqing	0.728	29.767	48.626	0.698	21.339	2665
	Average	0.669	24.967	50.329	0.652	43.234	3020
Hargreaves	Beijing	0.659	21.534	51.117	0.650	50.072	3156
	Hangzhou	0.693	25.461	53.913	0.665	36.779	3180
	Guangzhou	0.683	22.481	40.661	0.673	42.975	3047
	Harbin	0.618	22.622	54.003	0.607	55.754	2981
	Wuhan	0.659	23.908	51.761	0.686	52.406	3088
	Chongqing	0.744	26.345	47.135	0.696	22.075	2665
	Average	0.676	23.725	49.765	0.663	43.344	3020
BP neural network	Beijing	0.902	13.060	27.385	0.864	7.698	3158
	Hangzhou	0.894	18.316	31.648	0.854	7.498	3182
	Guangzhou	0.878	16.842	25.254	0.910	−4.069	3049
	Harbin	0.792	17.363	39.825	0.796	20.184	2983
	Wuhan	0.891	20.557	29.313	0.899	−0.245	3091
	Chongqing	0.912	20.084	27.717	0.862	8.983	2667
	Average	0.878	17.704	30.190	0.864	6.675	3022
Support vector machine	Beijing	0.899	13.192	27.851	0.861	7.882	3158
	Hangzhou	0.890	18.298	32.325	0.845	8.477	3182
	Guangzhou	0.881	17.139	24.889	0.919	−5.099	3049
	Harbin	0.791	17.470	39.939	0.787	20.197	2983
	Wuhan	0.894	20.770	28.865	0.933	−5.885	3091
	Chongqing	0.909	20.048	28.068	0.852	10.173	2667
	Average	0.877	17.820	30.323	0.866	5.958	3022

**Table 5:** Validation results of the UTCI calculation based on solar radiation estimated by empirical models and machine-learning methods.

Model	City	*NSE*	*MAPE*	*RMSE*	*Slope*	*Inter*	*n*
Angstrom	Beijing	0.993	7.683	1.121	0.994	−0.375	3157
	Hangzhou	0.990	7.210	1.137	0.992	−0.275	3181
	Guangzhou	0.989	3.788	0.907	1.001	−0.404	3048
	Harbin	0.987	10.162	2.013	1.017	−0.601	2982
	Wuhan	0.989	7.093	1.238	0.992	−0.309	3090
	Chongqing	0.989	4.505	0.998	0.983	0.175	2666
	Average	0.990	6.740	1.236	0.997	−0.298	3021
Ogelman	Beijing	0.993	7.617	1.112	0.994	−0.350	3157
	Hangzhou	0.991	6.453	1.044	0.993	−0.245	3181
	Guangzhou	0.990	3.584	0.843	1.003	−0.425	3048
	Harbin	0.987	10.034	1.994	1.018	−0.582	2982
	Wuhan	0.990	6.449	1.146	0.990	−0.213	3090
	Chongqing	0.990	4.331	0.967	0.975	0.308	2666
	Average	0.990	6.411	1.184	0.996	−0.251	3021
Bristow	Beijing	0.982	11.797	1.778	0.996	−0.134	3156
	Hangzhou	0.974	8.787	1.790	0.986	−0.144	3180
	Guangzhou	0.979	4.816	1.242	0.975	0.400	3047
	Harbin	0.981	12.933	2.366	1.006	−0.211	2981
	Wuhan	0.976	9.028	1.779	0.940	1.347	3088
	Chongqing	0.980	5.246	1.368	0.972	0.079	2665
	Average	0.979	8.768	1.721	0.979	0.223	3020
Hargreaves	Beijing	0.980	12.380	1.858	0.994	−0.178	3156
	Hangzhou	0.975	8.810	1.774	0.988	−0.219	3180
	Guangzhou	0.978	5.079	1.280	0.976	0.393	3047
	Harbin	0.983	12.607	2.297	1.005	−0.218	2981
	Wuhan	0.980	8.134	1.635	0.951	1.197	3088
	Chongqing	0.982	4.970	1.277	0.965	0.269	2665
	Average	0.980	8.663	1.687	0.980	0.207	3020
BP neural network	Beijing	0.994	7.024	1.036	0.992	−0.322	3158
	Hangzhou	0.993	5.496	0.944	0.996	−0.274	3182
	Guangzhou	0.992	3.008	0.774	0.999	−0.369	3049
	Harbin	0.988	9.361	1.878	1.011	−0.573	2983
	Wuhan	0.991	6.008	1.106	0.996	−0.326	3091
	Chongqing	0.992	3.206	0.874	0.983	0.137	2667
	Average	0.992	5.684	1.102	0.996	−0.288	3022
Support vector machine	Beijing	0.994	7.201	1.056	0.992	−0.343	3158
	Hangzhou	0.993	5.533	0.950	0.996	−0.284	3182
	Guangzhou	0.992	3.103	0.770	1.001	−0.391	3049
	Harbin	0.988	9.638	1.878	1.010	−0.597	2983
	Wuhan	0.992	5.576	1.038	0.993	−0.258	3091
	Chongqing	0.992	3.176	0.847	0.981	0.204	2667
	Average	0.992	5.705	1.090	0.996	−0.278	3022

**Table 6:** Detailed information on UTCI and the yearly days within each category in the large tourism cities of China.

Cities	UTCI (°C)	C3 (Day)	C4 (Day)	C5 (Day)	C6 (Day)	C7 (Day)	C8 (Day)	C9 (Day)
BJ	11.6 ± 1.3 [8.7,13.9]	7.2 ± 5.0 [0,22]	60.2 ± 8.7 [40,81]	146.8 ± 10.6 [122,171]	61.1 ± 11.1 [39,84]	74.2 ± 9.8 [53,95]	14.6 ± 8.8 [1,36]	1.2 ± 2.0 [0,8]
TJ	11.7 ± 1.3 [8.8,14.1]	8.6 ± 5.5 [0,24]	61.4 ± 10.1 [38,82]	142.9 ± 10.0 [113,165]	62.4 ± 10.2 [36,91]	74.6 ± 11.5 [53,102]	14.8 ± 8.3 [1,41]	0.9 ± 1.2 [0,5]
DL	5.5 ± 2.8 [0.3,10.8]	0.5 ± 1.8 [0,13]	24.2 ± 12.6 [3,53]	150.3 ± 11.8 [126,179]	56.2 ± 9.3 [35,85]	81.5 ± 10.5 [60,105]	40.0 ± 13.5 [9,66]	10.8 ± 7.3 [0,28]
QD	7.3 ± 2.3 [1.8,11.7]	0.8 ± 1.7 [0,9]	27.3 ± 13.0 [6,55]	154.2 ± 10.8 [135,178]	67.7 ± 10.7 [49,97]	77.9 ± 13.3 [46,102]	31.6 ± 10.7 [10,61]	5.3 ± 4.6 [0,19]
SH	14.2 ± 1.9 [10.2,17.8]	17.8 ± 10.2 [1,42]	54.1 ± 8.2 [37,66]	166.8 ± 17.1 [133,208]	72.6 ± 9.7 [50,93]	48.5 ± 15.7 [14,79]	5.3 ± 6.1 [0,28]	0.1 ± 0.3 [0,1]
NJ	15.4 ± 1.2 [12.8,18.3]	23.6 ± 9.8 [6,52]	57.7 ± 9.5 [35,80]	168.2 ± 13.4 [138,207]	70.4 ± 9.2 [53,91]	41.5 ± 12.2 [12,71]	3.2 ± 3.4 [0,15]	0.0 ± 0.0 [0,0]
SZ	14.8 ± 2.2 [10.1,18.5]	22.1 ± 11.4 [4,45]	54.3 ± 9.4 [33,79]	167.6 ± 16.1 [129,201]	72.6 ± 9.6 [56,91]	44.0 ± 19.5 [8,89]	4.1 ± 5.4 [0,22]	0.1 ± 0.3 [0,1]
HZ	16.8 ± 1.1 [14.5,18.8]	32.6 ± 12.1 [14,61]	56.3 ± 9.7 [25,82]	176.8 ± 13.5 [145,205]	68.0 ± 9.1 [48,95]	29.7 ± 10.6 [8,58]	1.5 ± 2.0 [0,10]	0.0 ± 0.0 [0,0]
XM	20.5 ± 1.3 [17.4,22.4]	28.5 ± 14.7 [3,66]	93.1 ± 13.8 [59,122]	198.6 ± 13.9 [169,232]	39.2 ± 13.0 [13,63]	5.8 ± 5.6 [0,24]	0.0 ± 0.0 [0,0]	0.0 ± 0.0 [0,0]
GZ	23.4 ± 0.9 [21.2,25.0]	48.7 ± 11.1 [21,71]	120.0 ± 13.0 [95,156]	171.8 ± 11.9 [149,200]	20.4 ± 7.1 [5,39]	4.3 ± 4.9 [0,18]	0.1 ± 0.2 [0,1]	0.0 ± 0.0 [0,0]
SE	23.5 ± 1.0 [21.5,26.0]	47.2 ± 12.4 [25,86]	122.5 ± 13.8 [100,160]	173.8 ± 15.2 [143,201]	18.0 ± 7.3 [3,34]	3.7 ± 3.6 [0,16]	0.0 ± 0.2 [0,1]	0.0 ± 0.0 [0,0]
SY	26.7 ± 3.0 [19.4,30.4]	66.7 ± 45.6 [0,153]	160.3 ± 33.2 [76,204]	134.2 ± 63.0 [46,272]	4.0 ± 8.1 [0,39]	0.2 ± 0.7 [0,4]	0.0 ± 0.0 [0,0]	0.0 ± 0.0 [0,0]
QD	9.9 ± 1.6 [5.7,12.7]	2.3 ± 3.2 [0,14]	44.6 ± 9.6 [16,66]	152.8 ± 9.9 [128,170]	59.5 ± 8.4 [45,81]	88.7 ± 9.3 [53,109]	16.5 ± 11.2 [1,45]	0.8 ± 1.6 [0,9]
NB	16.8 ± 1.5 [14.0,20.3]	31.0 ± 13.2 [6,60]	58.9 ± 9.1 [32,78]	177.8 ± 14.8 [146,209]	68.2 ± 10.1 [46,92]	27.6 ± 12.1 [4,50]	1.6 ± 2.2 [0,10]	0.0 ± 0.0 [0,0]
HB	0.5 ± 3.0 [−5.1,5.9]	0.3 ± 0.7 [0,3]	16.9 ± 10.0 [1,46]	129.3 ± 13.6 [101,163]	46.7 ± 8.1 [31,72]	62.4 ± 8.5 [47,90]	85.3 ± 11.2 [52,110]	23.3 ± 19.4 [1,72]
ZZ	13.9 ± 1.6 [9.7,17.4]	14.6 ± 7.7 [4,42]	64.3 ± 9.9 [40,86]	153.7 ± 11.0 [127,179]	69.9 ± 9.4 [45,91]	55.1 ± 12.8 [24,79]	7.1 ± 6.7 [0,30]	0.4 ± 1.1 [0,7]
WH	17.4 ± 1.6 [14.1,20.3]	36.8 ± 10.8 [14,60]	65.9 ± 10.4 [46,89]	164.5 ± 15.8 [126,209]	66.6 ± 9.6 [40,95]	27.4 ± 14.0 [2,61]	2.3 ± 3.2 [0,14]	0.1 ± 0.3 [0,2]
ZJ	18.3 ± 0.9 [16.5,19.8]	32.7 ± 9.9 [13,55]	68.9 ± 9.7 [51,90]	183.4 ± 14.0 [156,220]	65.3 ± 10.0 [44,95]	14.4 ± 9.6 [0,40]	0.5 ± 1.2 [0,7]	0.0 ± 0.0 [0,0]
CS	16.7 ± 0.9 [14.8,19.4]	37.6 ± 10.5 [18,73]	64.0 ± 8.0 [49,86]	161.7 ± 13.2 [129,195]	61.3 ± 9.6 [33,84]	36.7 ± 9.5 [16,60]	3.5 ± 3.0 [0,12]	0.1 ± 0.3 [0,2]
HS	−1.7 ± 1.7 [−4.8,1.3]	0. ± 0.1 [0,1]	0.1 ± 0.3 [0,2]	91.6 ± 17.3 [61,125]	92.9 ± 11.1 [62,121]	101.6 ± 8.9 [80,120]	62.7 ± 10.5 [43,85]	14.8 ± 6.1 [1,30]
GL	18.2 ± 1.2 [15.5,20.2]	36.0 ± 9.3 [13,59]	89.0 ± 9.7 [65,117]	156.7 ± 12.6 [122,190]	48.9 ± 7.6 [30,67]	30.8 ± 10.2 [6,51]	3.9 ± 4.3 [0,16]	0.1 ± 0.2 [0,1]
CC	1.3 ± 2.5 [−4.5,6.4]	0.4 ± 0.8 [0,4]	17.8 ± 10.0 [3,44]	131.9 ± 10.7 [106,158]	46.3 ± 8.3 [27,66]	68.1 ± 12.1 [41,91]	79.9 ± 11.7 [52,107]	20.3 ± 15.5 [0,65]
HH	6.9 ± 1.8 [2.4,9.2]	0.2 ± 0.6 [0,3]	18.4 ± 9.6 [0,41]	162.9 ± 12.0 [134,186]	59.3 ± 8.7 [34,81]	87.7 ± 11.5 [65,109]	33.4 ± 15.8 [9,74]	3.4 ± 3.2 [0,16]
JZ	8.9 ± 1.3 [5.7,11.5]	0.2 ± 0.6 [0,3]	26.5 ± 8.7 [7,48]	169.2 ± 10.4 [152,199]	60.4 ± 9.6 [40,80]	89.0 ± 9.7 [64,110]	18.8 ± 9.1 [2,41]	1.1 ± 1.3 [0,5]
NC	17.7 ± 1.7 [14.5,20.4]	45.8 ± 10.3 [22,74]	64.0 ± 11.4 [38,87]	162.3 ± 14.8 [128,198]	57.0 ± 8.5 [36,75]	29.8 ± 12.9 [7,59]	4.6 ± 6.0 [0,24]	0.3 ± 0.7 [0,4]
XA	14.5 ± 1.1 [12.6,18.0]	11.7 ± 6.9 [0,29]	57.2 ± 9.0 [37,78]	172.0 ± 13.3 [150,197]	80.5 ± 10.6 [57,103]	41.9 ± 12.2 [18,73]	2.0 ± 2.5 [0,13]	0.0 ± 0.0 [0,0]
CQ	19.0 ± 0.8 [17.1,20.5]	33.5 ± 10.5 [13,58]	63.2 ± 11.6 [41,94]	203.1 ± 12.4 [179,234]	62.7 ± 12.6 [37,97]	2.0 ± 2.6 [0,14]	0.0 ± 0.0 [0,0]	0.0 ± 0.0 [0,0]
CD	17.5 ± 0.7 [16.4,20.1]	6.6 ± 5.4 [0,21]	65.7 ± 8.8 [50,101]	216.3 ± 15.0 [176,243]	72.2 ± 12.2 [45,105]	4.4 ± 3.9 [0,22]	0.0 ± 0.1 [0,1]	0.0 ± 0.0 [0,0]
KM	15.8 ± 1.3 [13.4,18.5]	0. ± 0. [0,0]	1.7 ± 2.3 [0,10]	319.2 ± 18.4 [274,350]	39.0 ± 17.4 [11,78]	5.3 ± 3.3 [0,13]	0.1 ± 0.2 [0,1]	0.0 ± 0.1 [0,1]
LJ	11.3 ± 1.6 [8.6,14.0]	0. ± 0. [0,0]	0.1 ± 0.4 [0,2]	226.2 ± 27.7 [185,279]	113.1 ± 14.3 [83,140]	25.7 ± 19.9 [1,75]	0.1 ± 0.3 [0,2]	0.0 ± 0.0 [0,0]
ZY	16.5 ± 0.8 [15.1,18.4]	3.4 ± 4.6 [0,18]	64.6 ± 12.9 [39,93]	204.9 ± 17.8 [175,245]	78.2 ± 11.5 [45,111]	14.2 ± 8.2 [1,35]	0.0 ± 0.2 [0,1]	0.0 ± 0.0 [0,0]
YC	10.1 ± 1.1 [7.5,12.5]	0.7 ± 1.3 [0,7]	32.3 ± 8.9 [9,51]	171.0 ± 10.5 [151,199]	64.8 ± 9.4 [40,89]	84.4 ± 11.4 [55,113]	11.9 ± 6.3 [1,28]	0.2 ± 0.4 [0,1]
JQ	7.4 ± 1.3 [4.6,10.1]	0. ± 0.1 [0,1]	13.1 ± 7.0 [0,32]	168.2 ± 7.7 [155,186]	64.6 ± 9.0 [46,84]	97.9 ± 10.8 [68,122]	21.1 ± 10.7 [5,45]	0.3 ± 0.5 [0,2]
XN	7.7 ± 1.6 [3.8,9.9]	0. ± 0. [0,0]	0.9 ± 1.9 [0,8]	182.9 ± 15.5 [136,208]	86.0 ± 11.2 [54,117]	86.5 ± 13.4 [59,116]	8.9 ± 8.3 [0,29]	0.1 ± 0.4 [0,2]
WL	6.5 ± 1.5 [1.6,9.0]	0.5 ± 1.3 [0,7]	21.8 ± 9.9 [3,42]	160.2 ± 11.5 [139,198]	50.5 ± 8.6 [36,71]	85.9 ± 14.0 [57,118]	45.1 ± 14.0 [13,75]	0.1 ± 2.0 [0,9]

Note: In A ± B [X,Y], A, B, X, and Y denote the mean, standard deviation, minimum value, and maximum value, respectively.

## Data Availability

Not applicable.
